# Self-Consistent Examination of Donachie's Constant Initiation Size at the Single-Cell Level

**DOI:** 10.3389/fmicb.2015.01349

**Published:** 2015-12-08

**Authors:** Sattar Taheri-Araghi

**Affiliations:** ^1^Department of Physics, University of California, San DiegoLa Jolla, CA, USA; ^2^Department of Physics and Astronomy, California State University, NorthridgeNorthridge, CA, USA

**Keywords:** cell size, adder principle, cell cycle, chromosome replication, critical initiation size, single-cell analysis

## Abstract

How growth, the cell cycle, and cell size are coordinated is a fundamental question in biology. Recently, we and others have shown that bacterial cells grow by a constant added size per generation, irrespective of the birth size, to maintain size homeostasis. This “adder” principle raises a question as to when during the cell cycle size control is imposed. Inspired by this question, we examined our single-cell data for initiation size by employing a self-consistency approach originally used by Donachie. Specifically, we assumed that individual cells divide after constant C + D minutes have elapsed since initiation, independent of the growth rate. By applying this assumption to the cell length vs. time trajectories from individual cells, we were able to extract theoretical probability distribution functions for initiation size for all growth conditions. We found that the probability of replication initiation shows peaks whenever the cell size is a multiple of a constant unit size, consistent with the Donachie's original analysis at the population level. Our self-consistent examination of the single-cell data made experimentally testable predictions, e.g., two consecutive replication cycles can be initiated during a single cell-division cycle.

## 1. Introduction

The coordination between growth and the cell cycle is a fundamental aspect of cellular physiology. The classic work of Schaechter, Maaløe and Kjelgaard established the “growth law,” which states that the average size of bacterial cells in steady-state growth condition scales exponentially with the respective average growth rate (Schaechter et al., 1958). This is one of the first quantitative principles in bacterial physiology. Another important quantitative principle is the bacterial cell cycle model, whose two cornerstone assumptions are (i) in balanced growth the duration of replication (C period) of *Escherichia coli* chromosome is constant independent of the growth condition and (ii) cell divides after a constant time (C + D period) has elapsed since replication initiation (Cooper and Helmstetter, 1968; Helmstetter, 1968; Cooper, 1969).

In an important work, Donachie studied the consequences of the growth law and the cell cycle model together (Donachie, 1968). He concluded that, if both models are correct, the size of the cell per origin at the moment replication is initiated should be constant for all growth conditions. Furthermore, if the two models are correct, then the growth law can be expressed using the measured C + D as

(1) $$\begin{array}{l} m\left(T\right)=m_{0}2^{\left(C+D\right)/T}, \end{array}$$

where *m* is the average cell size and *T* is average cell doubling time. In other words, Donachie was able to make experimentally testable predictions by self-consistently examining the relationship between two different assumptions. Furthermore, conversely, the predicted relationship can be used to estimate C + D using size *m* and the average doubling time *T*, which can be measured and tested independently. In Appendix A, we present another example of self-consistency check, i.e., by self-consistently combining the initiator model (Cooper, 1969; Helmstetter, 1969) and the cell cycle model, we can show that the growth law emerges.

In recent years, single-cell experiments have significantly improved our understanding of growth and cell-size control in bacteria [For a review see Taheri-Araghi et al. (2015b) and discussions therein]. Single-cell data reveal information about fluctuations, heterogeneity and correlations between measurable parameters, which are masked in population measurements. In particular, we and others have shown that bacteria employ an “adder” principle to maintain size homeostasis during steady-state growth (Campos et al., 2014; Taheri-Araghi et al., 2015a). That is, cells grow by a constant size from birth to division, irrespective of the birth size. This automatically ensures that deviations in cell-size are corrected within a few generations. The adder principle however raises an important issue of when during the cell cycle size control is imposed.

This work presents a single-cell version of Donachie's analysis to our data in Taheri-Araghi et al. (2015a). We assume that C + D is constant for all cells. Using this assumption, we retrace C + D minutes backward in time from each cell division to extract a hypothetical initiation size of individual cells. We then ask if these assumptions lead to constant initiation size at the single-cell level. We found that, if the C + D period is indeed constant for all cells, the constant initiation size is consistent with the adder principle at the single-cell level. Another prediction of our self-consistent analysis is that a cell can initiate two rounds of replication between birth and division. These predictions can be tested experimentally to verify the validity of the assumptions.

## 2. Materials and methods

### 2.1. Experimental data on growth and division of *E. coli*

We used experimental data of cell length vs. time for seven different growth conditions for *E. coli* reported in Taheri-Araghi et al. (2015a). The media, average generation time, and average newborn size of cells are listed in Table 1. For the details of the experiments and growth media see Taheri-Araghi et al. (2015a) and its Supplementary Material. For the details of the single-cell growth experiment see Wang et al. (2010).

**Table 1:** **Name of the growth conditions, average generation time, and average cell size at birth**.

**Name of growth media**	**Generation time (minutes)**	**Size at birth (*μm*^3^)**
TSB	17.1	2.73
Synthetic Rich	22.5	1.64
Glucose+12 a.a.	26.7	1.04
Glucose+6 a.a.	30.2	0.80
Glucose	37.7	0.59
Sorbitol	50.8	0.46
Glycerol	51.3	0.42

### 2.2. Retracing length vs. time data to infer initiation size

**Figure 1:**
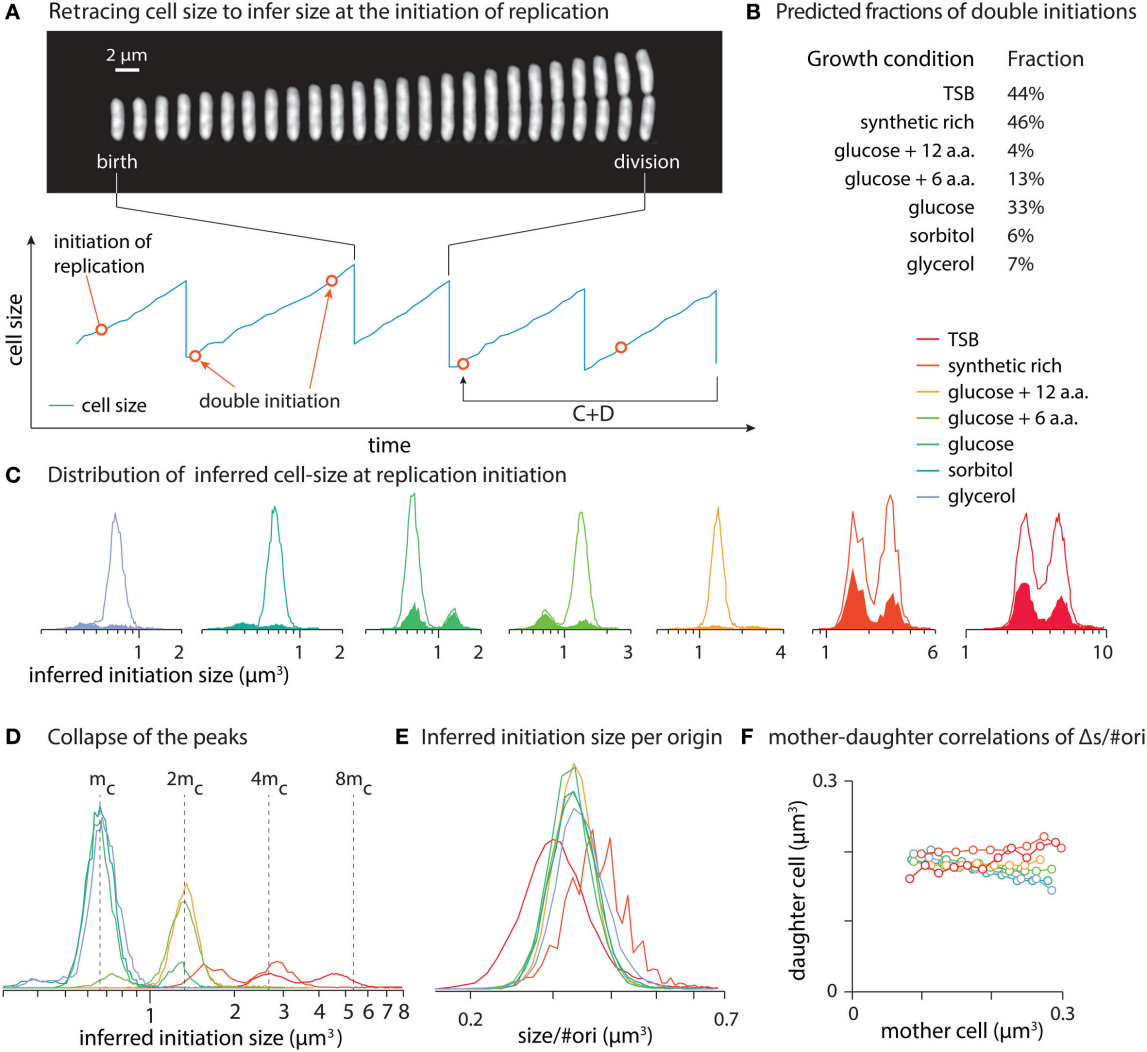
**(A)** Retracing cell size C + D minutes prior to cell divisions to infer size at initiation of replication. Constancy of C + D predicts some cells have two initiations in one division cell cycle. **(B)** Fractions of initiations that occur in generations with double initiations. **(C)** Distributions of hypothetical initiation size can be bimodal. Each panel refers to one growth condition where filled area show the distribution of double initiations and solid lines show the distribution of all hypothetical initiation sizes. **(D)** The peaks of the distributions in C collapse onto each other. **(E)** Inferred initiation size per origin of replication from various growth condition collapse onto each other. **(F)** Mother-daughter correlations of Δ*s*/#*ori* (growth per origin of replication), based on inferred initiation moments. Panel **(A)** is reproduced from Taheri-Araghi et al. (2015a) with permission from Elsevier.

We apply the cell cycle model by Helmstetter and Cooper (Cooper and Helmstetter, 1968; Helmstetter, 1968; Cooper, 1969) to infer the initiation size. That is, we assume that individual cells initiate replication C + D minutes prior to cell division (Figure 1A). We estimate C + D self-consistently by fitting the population average size vs. growth data from Taheri-Araghi et al. (2015a) to Equation (1). The fitting outcome is that C + D = 69 min.

**Figure A2:**
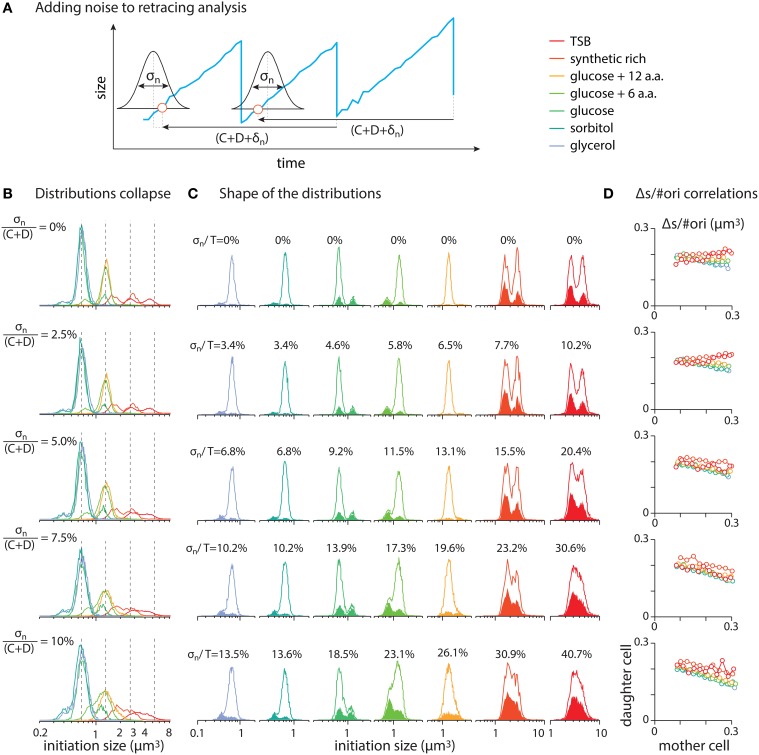
**(A)** To test the effect of noise in the analysis, a Gaussian noise δ_*n*_ with standard deviation of standard deviation of σ_*n*_ is added to the retracing time. **(B)** Various levels of noise is tested with standard deviations, σ_*n*_, chosen at 0, 2.5, 5, 7.5, and 10% of C + D. **(C)** Solid lines refer to the distribution from whole cell population and filled area refer to cells with double initiations in a division cycle. Standard deviation, σ_*n*_, in each row is the same as the one in the corresponding row in **(B)**. The ratio of the standard deviations, σ_*n*_, to the average doubling time, *T*, is noted on the left side of each sub panel. **(D)** Mother-daughter correlations of Δ*s*/#*ori* with noise added in retracing.

Since we do not have direct experimental data on the actual fluctuations of C and D periods, we cannot quantify the error arising from the retracing method. However, we can add noise to C + D extracted by fitting data to Equation (1), and use it to check robustness of our conclusions. In Appendix B, we present a detailed discussion on the effect of noise in C + D. We find that the predictions of our analysis are robust to random fluctuations in the C and D periods, unless the added noise is larger than ≳ 20% of the generation time (Figure A2).

We provide a final self-consistency check that our single-cell analysis agrees with the population level results in Appendix C.

## 3. Results and discussion

### 3.1. Distribution of inferred initiation size shows distinct peaks, consistent with donachie's constant initiation size model

We computed distributions of hypothetical initiation size by retracing the single-cell length vs. time data for seven different growth conditions (Figure 1B). All distributions showed peaks. An obvious question is whether these peaks are multiples of constant initiation size as Donachie inferred from population data. To answer this question, we overlaid the distributions (Figure 1D).

Indeed, we found that the peaks of the inferred initiation size distributions collapse onto each other, with the peak positions increase in exponent of 2 from the position of the left-most peak. We then calculated inferred initiation size per replication origin (Figure 1E). Distributions from various growth conditions collapse on each other in the form of single-peak distributions. This is consistent with the model that replication initiates whenever the cell size per origin reaches a constant critical size, regardless of the growth condition (Donachie, 1968; Pritchard, 1968). (In Appendix D we show how the number of replication origins is calculated.)

A prediction of our self-consistent analysis is the possibility of double initiations. For significant fractions of subpopulations of cells, retracing by constant C + D predicted two initiations separated by growth of a constant size per origin between them within a single generation (Figures 1B–E). This is not what is expected from the basic assumptions of the cell cycle control, which requires one-to-one correspondence between replication cycle and division cycle (Mitchison, 1971). Since this prediction seemingly violates a basic assumption, direct experimental test at the single-cell level will be important.

### 3.2. Conditions for consistency of constant (C + D) model with adder principle

Another important question is whether the Helmstetter-Cooper model based on constant C + D is consistent with the adder principle. The organized pattern of inferred initiation size in Figures 1D,E can support such consistency. Unfortunately, with our current data we cannot answer whether replication starts at a critical size or after the cell grows for a constant size per origin from previous initiation. However, we can test if the constant C + D assumption and the adder principle are consistent by mother-daughter correlations. In Figure 1F, we show that there are no significant correlations between the mother and the daughter cells in terms of added size per origin (Δ*s*/#*ori*), as expected by the adder principle. That is, growth of the daughter cell by a constant Δ*s*/#*ori* between initiation events is independent of the mother. Since Δ*s*/#*ori* has been estimated by the constant C + D assumption, our analysis suggest that the two assumptions are mutually consistent.

Growth by a constant size per origin is consistent with the classic initiator model by Helmstetter and Cooper, stating that chromosome replication starts once the accumulation of initiators reach a critical threshold level (Cooper, 1969; Helmstetter, 1969). A feedback mechanism was proposed by Sompayrac and Maaloe (1973) to maintain initiator level proportional to cell size. We showed in Appendix A how the initiator and the cell cycle model by Helmstetter and Cooper can lead to the growth law.

While the initiator model seems plausible for the coordination of cell size and the replication cycle, there are experimental data that cannot be explained by the initiator model. For example, it has been shown that both an ectopic origin and the original wildtype origin initiate simultaneously without significant changes in growth kinetics (Wang et al., 2011). Another example is synchronous replication of minichromosomes that carry similar origin of replication in cells (Messer et al., 1978; Leonard and Helmstetter, 1986). In these examples, the relationship between size and number of origins do not follow the wild-type. At this point, we do not have sufficient experimental evidence to confirm the initiator model and the critical size for initiation and its link to the adder principle. Nevertheless, one way to reconcile a consistency between adder and constant C + D is to have an adder-like behavior for cell size at the initiation of chromosome replication.

### 3.3. Future work

In this work, we applied Donachie's self-consistent analysis to the single cell data we reported recently. With the assumption that C + D is constant for individual cells, our analysis makes two predictions that can be directly tested experimentally in the future work: (i) double initiations of chromosome replication in one division cycle, and (ii) growth by a constant size between two consecutive replication initiations. Single-cell level test of these predictions will clarify whether our assumption of constancy of C + D is valid. Cell-size dependency or large fluctuations of C + D can change these predictions.

Several recent models discussed various size control routes in bacteria (Amir, 2014; Campos et al., 2014; Iyer-Biswas et al., 2014a,b; Kennard et al., 2014; Osella et al., 2014; Taheri-Araghi et al., 2015a). An interesting, unresolved question is how size control principles align with the cell cycle control. For a conclusive answer, we need direct experimental data on the progression of cell cycle in individual cells.

Finally, while adder principle appears general for all bacterial organisms tested so far, eukaryotes are not perfect adder (Jun and Taheri-Araghi, 2015). Further insights on the molecular mechanism of the adder principle can be gained through experimental tests in which we can perturb the perfect adder. Previously, perturbation of cell division machinery has been experimentally linked to variations of cell size (Weart et al., 2007; Chien et al., 2012; Hill et al., 2013). The timing of replication initiation was also linked to cell size, where *E. coli* mutants of smaller size delay initiation until they reach the appropriate initiation size (Hill et al., 2012). Interestingly, a modest over expression of DnaA-ATP can recover the replication initiation timing. We believe experiments on wild-type or size mutants in which the rate of accumulation of possible initiators can be temporarily decoupled from cell size (with overexpression or inhibition of their expression) will reveal valuable information on the regulation of cell size and the coordination of the cell cycle with cell size.

## Funding

This work was supported by Paul G. Allen Foundation, the Pew Charitable Trusts, and the National Science Foundation CAREER Award (MCB-1253843) to Suckjoon Jun.

### Conflict of interest statement

The author declares that the research was conducted in the absence of any commercial or financial relationships that could be construed as a potential conflict of interest.

## Figures and Tables

**Figure A1:**
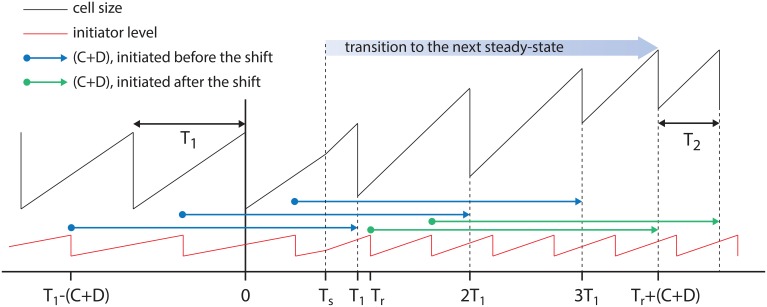
**Cell cycle and nutrient shift**. Here *n* = 3 cell cycles overlap at the moment of nutrient shift, *t* = *T*_*s*_. In this example *T*_1_ > *T*_2_ and thus *m*(*T*_1_) < *m*(*T*_2_). The three rounds of cell cycle started before nutrient shift lead to divisions at *T*_1_, 2*T*_1_, and 3*T*_1_ (blue arrows). The first initiation of replication after the nutrient shift, sets up the start of the new steady-state (green arrows starting at *T*_*r*_).

**Figure A3:**
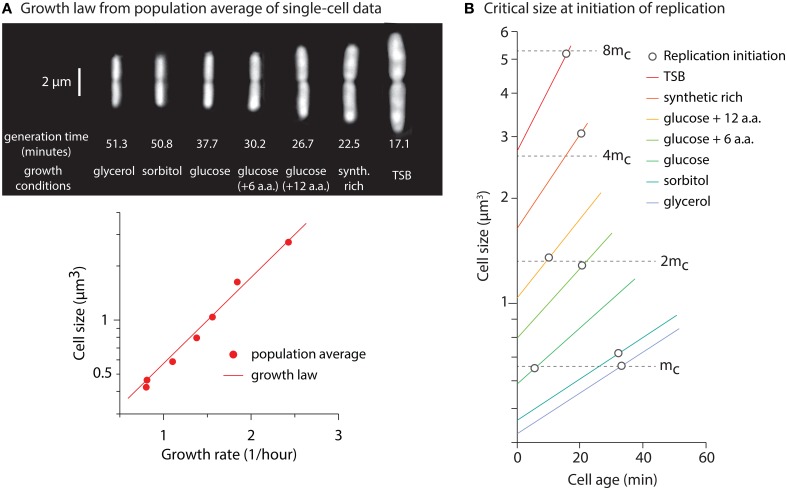
**(A)** (Top panel) Typical dividing cells of an isogenic *E. coli* strain under various growth conditions. (Bottom panel) To extract C + D, we fit $m\left(T\right)=m_{0}2^{\left(C+D\right)/T}$ to population average data. Here, growth rate is defined as ln(2)/*T*. **(B)** Self-consistency check of Donachie's critical size in our data. Each line shows average cell-size from birth to division as a function of cell age. The circle markers refer to initiation of replication, calculated by retracing average cell size C + D minutes before each division. Panel **(A)** is reproduced from Taheri-Araghi et al. (2015a) with permission from Elsevier.

**Figure A4:**
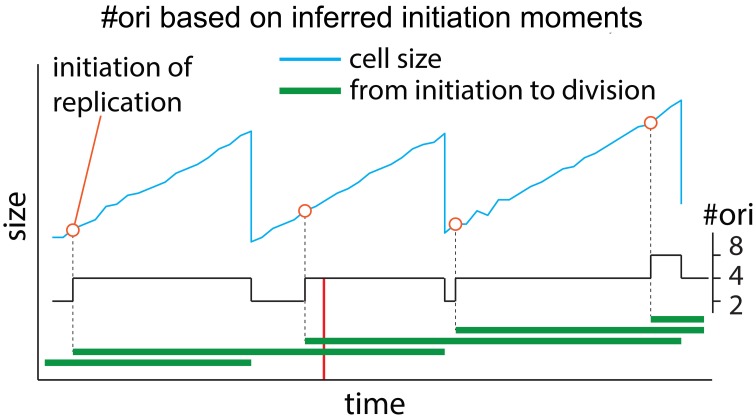
**Number of origins of replication, #***ori***, based on inferred initiation moments**. If *N* replication cycles overlap at any time point (green horizontal lines in the graph) during cell cycle, the total number of origins at that time point is #*ori* = 2^*N*^.

**Figure A5:**
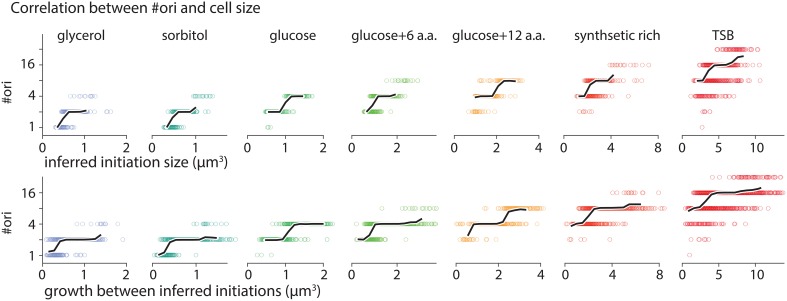
**Correlation between #***ori*** and cell size**. Number of origins, #*ori*, as a function of cell size at inferred initiation points (top panels) and as a function of growth between two consecutive initiations (bottom panel). #*ori* is a discrete parameter taking only values in exponents of two (#*ori* = 1, 2, 4,…). However, it shows a positive correlation with cell size. Black lines present *average #ori* as a function of inferred initiation size and growth between replication initiations.

## Appendix A

### Consistency between the initiator model, growth law and critical initiation size

In this appendix we model a nutrient shift experiment where the timing of the initiation of chromosome replication and, thus, cell divisions are calculated based on the initiator model proposed and tested by Helmstetter and Cooper (Cooper, 1969; Helmstetter, 1969). We derive an analytical solution for cell size and the cell cycle of bacteria that experience a nutrient shift. From that, we show that the growth law and the critical size model emerge from the initiator model.

Below is the list of the assumptions of the model:

- Chromosome replication initiates once the initiators accumulate to a critical level. When a round of replication starts, initiators get destroyed.
- There is a constant time gap, *C* + *D*, between each initiation of replication and cell division.
- During steady-state and nutrient-shift the timing of initiations of replication are given by:
  - In steady-state: period of initiators accumulation up to the threshold is *T*, equivalent to cells' generation time.
  - During nutrient shift from doubling times *T*_1_ to *T*_2_: If shift occurs at *t*_1_ minutes after the last initiation, next initiation happens at *t*_1_ + *t*_2_, where *t*_1_/*T*_1_ + *t*_2_/*T*_2_ = 1 (Cooper, 1969).
- The cell grows exponentially and the size increase rate changes promptly upon a nutrient shift.

To begin with, consider a cell growing in steady-state condition with doubling time *T*_1_. At time *T*_*s*_ after a division, the nutrient condition changes. The new condition imposes an eventual doubling time of *T*_2_ in the final steady-state. Here we choose the reference of the time, *t* = 0, the birth of the cell in which nutrient shift happened (Figure A1). Thus, nutrient shift happens at *t* = *T*_*s*_ during a cell cycle that the cell was expected to divide at *t* = *T*_1_. The time of planned division at *T*_1_ can change or remain unchanged depending on the timing of *T*_*s*_ with respect to rounds of chromosome replications. We take three steps to proceed with the calculation:

- We find the timing of initiations of chromosome replication, both before nutrient shift and after nutrient shift.
- From the timing of replication initiations, we find timing of cell divisions, assuming every initiation results in a cell division after *C* + *D* minutes.
- From the timing of cell division we calculate cell size considering that size increases exponentially with a rate instantaneously proportional to nutrient condition.

Since multiple cell cycles can overlap in bacteria, we assume at the time of nutrient shift, *T*_*s*_, there are *n* cell cycles overlapping (*n* = 0, 1, 2, …). The case *n* = 0 refers to the cells in slow growth condition if nutrient shift happens in the gap between the birth and initiation of chromosome replication (B period). There is a relationship between *n*, *T*_1_, *T*_*s*_, and *C* + *D*, the shorter the *T*_1_ the larger the *n* can be. Also, *n* can vary depending on when the nutrient shift happens during the cell cycle. Without loss of generality, we choose not to elaborate further on this relationship as the final results can be expressed in terms of *T*_1_, *T*_2_, and *C* + *D*.

#### Timing of chromosome replications

Since there is a one-to-one correspondence between initiation of replication and cell division, the time gap between the *n* rounds of ongoing cell cycles at *t* = *T*_*s*_ must be the generation time in the pre-shift condition, *T*_1_. As the first cell division after *t* = 0 was scheduled at *T*_1_, the “oldest” of the *n* overlapping cell cycles must have started at *T*_1_ − (*C* + *D*). The rest of them started every *T*_1_ minutes thereafter. The initiation times of these *n* cell cycles are, thus, the following series:

(A1) $$\begin{array}{l} T_{1}-\left(C+D\right),2T_{1}-\left(C+D\right),\ldots ,nT_{1}-\left(C+D\right). \end{array}$$

Let's consider the next chromosome replication (the first after nutrient shift) starts at *t* = *T*_*r*_, which depends on both time of nutrient shift, *T*_*s*_, and generation time in the second growth condition, *T*_2_. Next rounds of replication after *T*_*r*_ initiate every *T*_2_ minutes. Thus, the timing of replication initiation after the nutrient shift is simply:

(A2) $$\begin{array}{l} T_{r},T_{r}+T_{2},T_{r}+2T_{2},\ldots \end{array}$$

The time *T*_*r*_ can be calculated based on the third assumption of the model that we discussed earlier. Until the moment of nutrient shift, *T*_*s*_, initiators have been accumulating since the start of the last round of replication, *t* = *nT*_1_ − (*C* + *D*), with the rate of 1/*T*_1_. [e.g., 3*T*_1_ − (*C* + *D*) in Figure A1] From *t* = *T*_*s*_ until *t* = *T*_*r*_, initiators accumulate at rate 1/*T*_2_. The following equation can be solved for *T*_*r*_,

(A3) $$\begin{array}{l} \frac{1}{T_{1}}\left[T_{s}-nT_{1}+\left(C+D\right)\right]+\frac{1}{T_{2}}\left(T_{r}-T_{s}\right)=1, \end{array}$$

which yields

(A4) $$\begin{array}{l} T_{r}=\left(n+1\right)T_{2}+T_{s}-\frac{T_{2}}{T_{1}}\left(T_{s}+C+D\right). \end{array}$$

#### Timing of cell divisions

Each initiation of chromosome replication leads to a cell division after a time gap of (*C* + *D*). From Equations (A1) and (A2) and Figure A1, the cell division times from ongoing cell cycles and the ones starting after nutrient shift are:

(A5) $$\begin{array}{l} \underset{\text{divisions from ongoing cell cycles}}{\underset{︸}{T_{1},2T_{1},\ldots ,\left(n-1\right)T_{1},nT_{1},}}\\ \underset{\text{divisions from cell cycles that start after nutrient shift}}{\underset{︸}{T_{r}+\left(C+D\right),T_{r}+\left(C+D\right)+T_{2},T_{r}+\left(C+D\right)+2T_{2},\ldots }} \end{array}$$

#### Cell-size after the nutrient shift

Let's consider *m*(*T*_1_) denotes the newborn cell size during steady-state growth with generation time *T*_1_. We aim to calculate *m*(*T*_2_), the newborn cell size at steady-sate with generation time *T*_2_. Cells reach steady-state after the division at *T*_*r*_ + (*C* + *D*), where the gap between cell divisions is *T*_2_ and the size increase rate is 1/*T*_2_. Thus, the cell size after that division is the newborn cell-size in the new steady state, *m*(*T*_2_).

From *t* = 0 until *t* = *T*_*s*_ cell size increases exponentially with rate 1/*T*_1_ (pre-shift rate). Upon nutrient shift, the rate of cell size increase changes instantaneously to 1/*T*_2_. The division at *t* = *T*_*r*_ + (*C* + *D*) (corresponding to start of the new steady-state) is the (*n* + 1)*th* division after *t* = 0 (see Equation A5). Thus, the newborn cell size is given by

(A6) $$\begin{array}{l} m\left(T_{2}\right)=\frac{1}{2^{n+1}}m\left(T_{1}\right)2^{T_{s}/T_{1}}\times 2^{\left(T_{r}+C+D-T_{s}\right)/T_{2}}, \end{array}$$

where the first factor on the right-hand-side counts (*n* + 1) divisions, the second term refers to the growth from birth at *t* = 0 until nutrient shift at *t* = *T*_*s*_, and the third accounts for the size increase from the nutrient shift, *t* = *T*_*s*_, until division at *t* = *T*_*r*_ + *C* + *D*.

Substituting *T*_*r*_ from Equation (A4) in Equation (A6), we get

(A7) $$\begin{array}{l} m\left(T_{2}\right)=m\left(T_{1}\right)2^{-\left(C+D\right)/T_{1}}\times 2^{\left(C+D\right)/T_{2}} \end{array}$$

Equation (A7) denotes that if cells grow in any steady-state condition with generation time *T*, newborn cell size is exponentially related to *T*,

(A8) $$\begin{array}{l} m\left(T\right)=m_{\circ }2^{\left(C+D\right)/T}. \end{array}$$

This is the growth law (Schaechter et al., 1958), with the exponent being (*C* + *D*) if the relationship presented in the base of two. The exponent is consistent with Donachie's constant size at initiation of chromosome replication, as we elaborate below.

#### Cell-size at initiation of replication

Consider that cells growing in a steady-state condition with generation time *T* and that up to *N* cell cycles overlap (*N* = 1, 2, 3…). That means from an initiation of chromosome replication until the cell division (this corresponds to a gap of (*C* + *D*)) we have *N* cell division events. For slowest growth conditions where cell cycles do not overlap we have *N* = 1. If *m*_*c*_(*T*) refers to cell size at which initiation occurs, the newborn cell size after the corresponding cell division is

(A9) $$\begin{array}{l} \frac{1}{2^{N}}m_{c}\left(T\right)2^{\left(C+D\right)/T}=m\left(T\right), \end{array}$$

where the first factor accounts for *N* divisions and the rest accounts for growth for (*C* + *D*) minutes. Substituting *m*(*T*) from Equation (A8), we obtain

(A10) $$\begin{array}{l} m_{c}\left(T\right)=2^{N}m_{\circ }. \end{array}$$

Since 2^*N*^ is the total number of replication forks when *N* cell cycle overlap, Equation (A10) shows that cell size per origin of replication at the initiation of replication is constant, independent of growth condition. This is the Donachie's observation in 1968 by combining the growth law and Helmstetter-Cooper model.

## Appendix B

### Noise and uncertainty in retracing analysis

One may question the effect of noise in C + D in retracing analysis and the extent it influences the distributions reported in Figure 1. The retracing analysis has an intrinsic “reading error.” Since we do not have experimental data on the fluctuations of the durations of C and D periods for individual cells, we cannot completely avoid or quantify this reading error. However, in this appendix we present how we can choose a constant C + D to minimize the error. Here, we show that adding any noise to retracing time, C + D, merely adds *extra* reading error and does not capture actual fluctuations of C and D periods.

There are two different points that should not be mixed:

- The actual fluctuations in C and D periods.
- The noise that can be possibly added to retracing time, C + D.

The reading error, defined as the difference between the actual moment of initiation and the inferred moment, is a result of the combination of (i) and (ii). Regarding the point (i), we believe that there are fluctuations in C and D periods. However, to date, we do not have any experimental data measuring and addressing these fluctuations at a single-cell level. Regarding the point (ii), retracing is an indirect method of estimating initiation time and size. Adding noise to retracing time can test the extent the outcome is robust with respect to noise.

Let's consider the actual fluctuations of C + D periods (combined) have the standard deviation of σ_*a*_. In the case that we do not include noise in retracing time, the inferred readings miss these actual fluctuations. Thus, the readings error has the standard deviation of σ_*a*_ as well. Now consider that we add a noise in retracing time such that initiation times are estimated C + D + δ_*n*_ prior to cell divisions. Here δ_*n*_ is a stochastic variable. Let's assume the standard deviation of (C + D + δ_*n*_) is σ_*n*_. The noise δ_*n*_ is independent of fluctuations in C and D periods. Thus, this type of noise does not capture the actual fluctuations, but only adds more uncertainty to the reading of inferred initiation size and time.

If both actual fluctuations of C + D and added noise are Gaussian, we can calculate the standard deviation of the reading error. If the actual initiation points happen (C + D + δ_*a*_) prior to cell divisions, the retracing analysis reads those initiations at (C + D + δ_*n*_). (δ_*a*_ and δ_*n*_ are independent stochastic variables). Thus, the reading error is given by:

(A11) $$\begin{array}{l} error=\left(\text{C}+\text{D}+\delta_{\text{n}}\right)-\left(\text{C}+\text{D}+\delta_{\text{a}}\right). \end{array}$$

Since δ_*n*_ and δ_*a*_ are independent Gaussian variables, the standard deviation of the error is given by

(A12) $$\begin{array}{l} \sigma_{error}^{2}=\sigma_{n}^{2}+\sigma_{a}^{2}. \end{array}$$

Therefore, to minimize the error in retracing analysis, we should minimize the noise in C + D + δ_*n*_ in the analysis. For this reason using a constant C + D is a better choice for this study.

To visualize the effect of noise in the analysis, we added Gaussian noise in retracing time and tracked the changes in the distributions of the inferred initiation sizes. As expected, the noise widens the distributions and beyond certain points, it influences the bimodal shape of the distributions. Various levels of noise is tested with standard deviations, σ_*n*_, chosen at 0%, 2.5%, 5%, 7.5%, and 10% of C + D. Figure A2B, shows distributions of the initiation size for various level of noise, as noted on the left side of each panel.

Figure A2C depicts the distributions of inferred initiation size for individual growth conditions. The solid lines in each panel refer to the distributions from whole cell population and the filled area refer to cells with double initiations in one division cycle. The level of noise, σ_*n*_, in each row of Figure A2C is the same as the one in the corresponding row in Figure A2B. The numbers on top of each distribution show the ratio of the standard deviation, σ_*n*_, of the noise to the average doubling time in each growth condition, σ_*n*_/*T*. For noises with σ_*n*_/*T* ≳ 20%, the widening of the distributions are such that peaks of the bimodal distributions start to overlap. However, the prediction of double initiations are robust with respect to the shape of the distributions (see filled area in panels of Figure A2C).

In Figure A2D, we show the effect of added noise in the mother-daughter correlations of Δ*s*/#*ori*. We do not see significant correlations between mother and daughter cells. However, at high level of added noise, the correlations tends to slightly tilt toward negative values since a random noise affects the value of Δ*s*/#*ori* oppositely in two consecutive generations.

In conclusion, for the retracing analysis used in this work, a constant value for retracing time C + D minimizes the analysis error. Our test on the distributions of the inferred initiation cell size shows that the shape of distributions are more or less conserved if the noise in the retracing time is up to ~20% of the generation time. Beyond this threshold the shape of the distributions start to change.

## Appendix C

### Consistency check between population average and single-cell analysis

In this appendix, we reproduce the Donachie's analysis on the *average* cell-size data from our single-cell measurements. To this end, we use C + D extracted from fitting average data to Equation (1) (Figure A3A), where C + D = 69 min. As expected, applying retracing analysis reproduces Donachie's critical size model (Figure A3B). This analysis is solely for a secondary self-consistency check of critical size model on our single-cell data (i.e., given the method used here, we do not expect any result otherwise).

Figure A3B shows cell size from birth to division as a function of cell age for seven different growth conditions. The circle markers refer to the moment of the replication initiation. Since C + D is longer than the generation times for the growth conditions in the study, each initiation size essentially refers to cell size in previous generations. The initiation size of different growth conditions can be clustered such that the initiation size per replication origin is constant and independent of growth condition. For the slowest condition, $m_{c}=0.66\mu m^{3}$ and for the rest, critical initiation size increases by factors of two ^1^.

## Appendix D

### Calculating number of origin of replication based on initiation times

Based on inferred moments of initiation, one can mathematically calculate the number of origins during the cell cycle. A graphical example is presented in Figure A4. The graph shows cell size and the inferred moments of initiation as a function of time. The horizontal green lines show replication cycles from initiation of replications until the corresponding cell divisions. In slowest growth conditions, where we have either zero or one replication cycle going on in the cell (*N* = 0 or 1), we know that #*ori* = 1 or 2 (not shown in the figure). In fast growth conditions, *N* (>1) replication cycles can overlap. At any initiation of replication, number of overlapping cell cycles, *N*, increases by one and number of origins of replication, #*ori*, increases by a factor of two. At any cell division, number of overlapping cell cycles, *N*, decreases by one and #*ori* decreases by a factor of two. Thus, since for *N* = 0, #*ori* = 1 and for *N* = 1, #*ori* = 2 and since increasing *N* by one increases #*ori* by a factor of two, we conclude if *N* replication cycles overlap at any moment, #*ori* at that moment is given by #*ori* = 2^*N*^. For instance, at the moment shown with the red vertical line in Figure A4, *N* = 2 replication cycles overlap (two horizontal green lines cross the red line). Thus, the total number of #*ori* at that moment is 2^*N*^ = 4. This can be applied to any moment throughout the cell cycle to calculate #*ori* as a function of time.

To examine correlation between #*ori* and cell size, we plotted #*ori* calculated at inferred initiation points as a function of initiation cell size (Figure A5 top panels) and as a function of growth between initiations (Figure A5 bottom panels). #*ori* is a discrete quantity taking only values in exponents of two (#*ori* = 1, 2, 4,…). There is, however, a positive overall correlation between computed #*ori* and cell size as depicted by black lines in each panel of Figure A5 (black lines show *average #ori* as a function of corresponding size or growth). The positive correlation arises from the fact that size and generation time are negatively correlated and that shorter generation time results in more overlapping replication cycles, thus larger #*ori*. The parameter Δ*s*/#*ori* that is used in Figure 1F is calculated based on growth between inferred initiation points per #*ori* during that growth period.
